# Healthcare worker competencies for disaster training

**DOI:** 10.1186/1472-6920-6-19

**Published:** 2006-03-20

**Authors:** Edbert B Hsu, Tamara L Thomas, Eric B Bass, Dianne Whyne, Gabor D Kelen, Gary B Green

**Affiliations:** 1 Department of Emergency Medicine, Johns Hopkins University School of Medicine, Baltimore, MD, USA; 2 Department of Emergency Medicine, Loma Linda University School of Medicine, Loma Linda, CA, USA; 3 Department of Medicine, Evidence-based Practice Center, Johns Hopkins University School of Medicine, Baltimore, MD, USA

## Abstract

**Background:**

Although training and education have long been accepted as integral to disaster preparedness, many currently taught practices are neither evidence-based nor standardized. The need for effective evidence-based disaster training of healthcare staff at all levels, including the development of standards and guidelines for training in the multi-disciplinary health response to major events, has been designated by the disaster response community as a high priority. We describe the application of systematic evidence-based consensus building methods to derive educational competencies and objectives in criteria-based preparedness and response relevant to all hospital healthcare workers.

**Methods:**

The conceptual development of cross-cutting competencies incorporated current evidence through a systematic consensus building process with the following steps: (1) review of peer-reviewed literature on relevant content areas and educational theory; (2) structured review of existing competencies, national level courses and published training objectives; (3) synthesis of new cross-cutting competencies; (4) expert panel review; (5) refinement of new competencies and; (6) development of testable terminal objectives for each competency using similar processes covering requisite knowledge, attitudes, and skills.

**Results:**

Seven cross-cutting competencies were developed: (1) Recognize a potential critical event and implement initial actions; (2) Apply the principles of critical event management; (3) Demonstrate critical event safety principles; (4) Understand the institutional emergency operations plan; (5) Demonstrate effective critical event communications; (6) Understand the incident command system and your role in it; (7) Demonstrate the knowledge and skills needed to fulfill your role during a critical event. For each of the cross-cutting competencies, comprehensive terminal objectives are described.

**Conclusion:**

Cross-cutting competencies and objectives developed through a systematic evidence-based consensus building approach may serve as a foundation for future hospital healthcare worker training and education in disaster preparedness and response.

## Background

Although healthcare worker training has long been accepted as an integral part of disaster preparedness, traditional training practices have not been systematically developed, rigorously examined or objectively tested. Only in recent years, under the increased scrutiny of accelerated institutional and governmental preparedness efforts, has the emerging sciences of emergency preparedness and medical education converged and a body of evidence concerning effective practices for healthcare workforce education begun to arise. The development of higher standards for workforce education based on evidence-based practices, sound educational theory and quantitative outcome measures represents an important gap to be filled in national preparedness efforts.

Providing the estimated twelve million healthcare workers in the United States with effective disaster preparedness training poses several major challenges. First, best practices to be taught must be identified and recognized. Second, specific target audiences and the content they should be taught must be defined. Instructional content should be tailored to meet the training requirements for different job categories. Effective multidisciplinary disaster response demands acquisition and application not only of factual knowledge but also complex concepts, multi-level decision making, and specific technical skills. The evaluation of whether these skill sets have been efficiently conveyed and effectively acquired presents its own inherent challenges. Finally, differences among healthcare workers such as prior training, work experience, baseline abilities and cultural background directly impact training effectiveness and must be taken into consideration for the training of large groups. The application of a systematic, competency-based approach can provide the solid foundation to build a comprehensive program of training and evaluation required to meet these challenges.

As there is currently no accepted standard for healthcare worker training in disaster response, a number of programs have adopted different formats to achieve their stated training and educational goals. Several well-recognized courses and related training materials have been aimed at improving standardization and accessibility [1-4]. To date, these practical courses fulfill an important demand but have not yet been validated or incorporated as part of standard medical training. A recent systematic literature review noted the difficulty in drawing firm conclusions about the effectiveness of specific types of hospital disaster training due to the limited numbers of studies, marked heterogeneity of training methods and weaknesses in study design and evaluation [5]. In 2001, a consensus panel of disaster medicine experts strongly recommended that education on disasters should be formalized and evidence-based and that evaluation of education and interventions must be improved [6]. Given this rapidly developing field, a number of competencies and recommendations have been made public over the past several years for clinicians, hospital workers, hospital leaders, nurses, public health workers and, health professions students but each offer considerably less detail in terms of describing measurable objectives to conduct competency-based training [7-11]. The development of professional standards and educational programs based on both the evidence and sound educational theory remains an important gap to be filled. In this paper, we propose a competency-based approach with specific measurable objectives derived by a national expert panel as a paradigm for healthcare worker disaster preparedness and response training.

## Methods

This article focuses on the development of key competencies and terminal objectives for training of all healthcare workers in disasterpreparedness. A competency was defined as a complex combination of knowledge, attitudes, and skills demonstrated by individuals that are critical to the effective and efficient function of an organization [12]. A terminal objective was defined as the demonstrable performance following the completion of a set of instruction.

The development of competencies for healthcare worker disaster response training and education incorporated an evidence-based consensus building process with the following steps: (1) review of literature on relevant content areas; (2) structured review of existing competencies, national level courses and published training objectives; (3) synthesis of new cross-cutting competencies; (4) expert panel review; (5) refinement of new competencies and; (6) development of testable terminal objectives for each competency using similar processes covering requisite knowledge, attitudes and skills.

### Literature search

A literature search updated in January 2006 identified all peer-reviewed articles related to educational competencies for healthcare workers in disasters. This consisted of identifying sources, developing a search strategy for each source and conducting and documenting the search. Electronic database searching of PubMed and the Excerpta Medica database (EMBASE) used key terms including *disaster, mass casualty, training, education, course *and *competencies*. In addition, team members conducted a hand search of the literature focusing on reference lists of relevant reviews to ensure comprehensiveness.

### Structured review of existing competencies and courses

The scope of this process involved abstracting, reviewing and cataloguing existing competencies and courses in training healthcare workers for disaster preparedness. An internet search with the terms *disaster, mass casualty, MCI, chemical, biological, radiological, CBRNE, NBC, WMD, hazmat*, merged with *training, education, course *and *competencies *was conducted in January 2006 to identify existing competencies or courses for healthcare workers in disasters outside of the peer-reviewed literature. Each course or competency set was then abstracted and reviewed by the study team to determine whether it directly pertained to disasters, whether it addressed healthcare workers and whether there was an established partnership/ endorsement by a national professional organization or governmental agency. Additional relevant texts, manuals and regulatory requirements on training of hospital staff for disaster preparedness cited by these courses or published competency sets were reviewed. Courses or competency sets that did not include training for disaster response, did not target healthcare workers, or were solely sponsored by local organizations or institutions were excluded.

### Synthesis of new competencies

**Figure 1:**
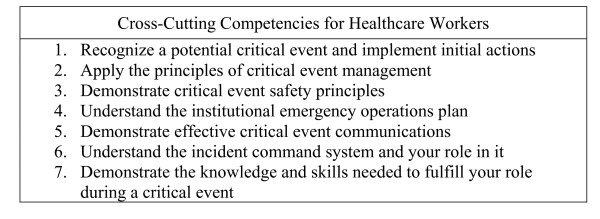
Cross-cutting competencies for healthcare workers.

Current educational theory on training, curriculum and competency development was reviewed with recognized educational experts and incorporated into the development process [13-15]. Each existing competency set was reviewed for appropriateness to the target audience, defined as all healthcare workers. The core concepts from each existing competency set were identified and abstracted with grouping of common elements. A new set of cross-cutting competencies and terminal objectives were drafted and internally reviewed by the team. (Figure 1) The term, "cross-cutting", is used here to describe the competencies which may be applied to related, but distinct target audiences within the field of healthcare workers. Such groups may include, but are not limited to first receiver physicians, first receiver nurses, other first receiver staff, critical event leadership, non-first receiver physicians, nurses, technical support staff and administration.

### Expert panel review

The team identified 12 nationally recognized experts, which included broad representation from relevant professional organizations, hospitals, academic centers, and government agencies. Two forms were completed by the expert panel including a competency assessment form and a terminal objectives assessment form.

The competency assessment form included questions designed to address clarity, appropriateness and relative importance of each of the draft competencies. It was also designed to elicit any other possible competency areas that had not been considered. Each response was assigned a score of one (strongly agree) to five (strongly disagree). The scores in each category from the respondents were averaged. Through the process, each draft competency scoring an average of 2.5 or less in all categories was selected with minor modifications, while any scoring over an average of 2.5 in any category was to undergo revision and be resent to the expert panel for additional comment. Specific expert panel comments were reviewed, discussed and incorporated by the team into the new competencies.

The terminal objectives assessment form was designed to address the clarity and relevance of each proposed terminal objective. Each respondent was asked to complete the form, rating the clarity and relevance of a particular terminal objective for the specified target audience from one (strongly agree) to five (strongly disagree). The scores in each category from the respondents were averaged. Each draft terminal objective scoring an average of 2.5 or less for clarity was selected with minor modifications. In contrast to the cross-cutting competencies, not all terminal objectives were designed to be relevant to the full spectrum of healthcare workers. Thus, the scores for relevance were used to identify the specific terminal objectives that should be restricted to certain target audiences (ie. those that are not relevant to all healthcare workers). As with the draft competencies, specific expert panel comments on the proposed terminal objectives were reviewed, discussed and incorporated by the team.

### Refinement of new competencies

Incorporating expert panel feedback through a modified Delphi process, the team developed and refined the final competencies and terminal objectives. These competencies and terminal objectives form the framework for healthcare worker critical event preparedness training, including determination of the content, training models and teaching techniques best suited for job specific training.

## Results

The literature search revealed relatively few articles describing educational competencies for training of healthcare workers in disaster preparedness and response. Several described the development process and competencies for health professions students, public health workers and, nurses in emergency response [11,16-24]. However, no articles were identified that outlined disaster training competencies with specific measurable objectives. Outside of the peer-reviewed literature, the structured review of existing courses and competencies identified six sets of published competencies pertaining to the disaster training among different types of healthcare professionals, including public health workers, medical students, first receivers and registered nurses [25-30]. In spite of their importance, none included specific measurable objectives in addressing the broader spectrum of healthcare workers.

Following review and synthesis of the core concepts from the published competencies described above, seven new draft competencies were evaluated by the expert panel. No additional competency areas were suggested by the expert panel. Expert panel opinion in the form of comments and scores ranging from one (strongly agree) to five (strongly disagree) were compiled. For clarity, each of the proposed competencies averaged a score of 2.8 or less (range 1.4 – 2.8). Regarding the relevance for the defined target audience, each of the proposed competencies scored 2.1 or less (range 1.4 – 2). In terms of importance, each of the proposed competencies scored 2.5 or less (range 1.0 – 2.5). For clarity, each of the proposed terminal objectives averaged a score of 2.5 or less (range 1.2 – 2.5). The scores for relevance of the proposed terminal objectives ranged from 1.1 – 3.6.

Through this process, a total of seven cross-cutting competencies and twenty-one terminal objectives for critical event training of all healthcare workers were developed. Since disaster training must integrate multi-disciplinary training with different levels of training, a cross-cutting competency approach can serve as a valuable starting point. Cross-cutting competencies can form the basis for standardized training and unify the disciplines and skill levels involved.

### Competency 1

#### Recognize a potential critical event and implement initial actions

The term, critical event, denotes any situation which threatens to disrupt the ability of an organization to maintain continuity of operations. Critical events include, but are not limited to disasters and emerging infectious diseases. An essential component of appropriate disaster response is the ability for the healthcare worker to recognize a critical event and know what to do – specifically who should be notified and how a disaster plan is activated. It is vital to provide early event recognition and early response mobilization in order to minimize the damage of the critical event. In addition, they should recognize triggers that precipitate reporting to the appropriate personnel.

#### TO1.1 – (Recognition)

Given scenarios that may be encountered in the course of normal professional duties, the participant should be able to identify all potential critical events and their event type. *To be successful, the participant must correctly identify all potential critical events among a list of scenarios*.

#### TO1.2 – (Notification)

Given a potential critical event scenario, the participant should be able to identify the appropriate authorities to be notified, recognize the appropriate notification steps and identify the key information to be reported. *To be successful, the participant must correctly identify the appropriate notification steps, information to be reported and correct reporting authority*.

#### TO1.3 – (Protection)

Given a description of a specific potential critical event, the participant should be able to list the immediate actions needed to protect personal, environmental and public safety. *To be successful, the participant must correctly identify standard safety precautions as well as additional precautions that may be needed for potential chemical, biological and radiological events*.

#### T O1.4 – (Mobilization)

Given a specific critical event scenario, the participant should be able to make recommendations for emergency response needs prior to disaster plan activation (mobilization). *To be successful, the participant must identify the specialized personnel and equipment that may be needed for the type of event and the preparation steps required for mobilization*.

#### TO1.5 – (Synthesis)

Given a simulated workplace scenario, the participant will apply knowledge of potential critical event recognition and immediate response needs to perform the appropriate notification, safety and mitigation actions for that event. *To be successful, the participant must correctly identify the type of potential critical event, identify the appropriate safety precautions for that event type and perform the appropriate simulated notification and mobilization actions*.

### Competency 2

#### Apply the principles of critical event management

In order for a facility to successfully manage all critical events, healthcare workers should understand the essential elements of an effective preparation and response including the appropriate actions to be performed.

#### TO2.1 – (Management)

Given a list of disaster terms and management activities, the participant will be able to identify the phases of critical event management and match the activities to the appropriate phase. *To be successful, the participant must be able to correctly recognize the phases from among a list of disaster terms and match the activities to the correct phase*.

#### TO2.2 – (Preparedness)

Given a critical event scenario, the participant will be able to apply their knowledge of disaster preparedness to identify the key components of preparedness and recognize appropriate preparedness activities. *To be successful, the participant must be able to correctly identify the components of disaster preparedness, and select the appropriate preparedness activities for each preparedness component*.

#### TO2.3 – (Response)

Given a critical event scenario, the participant will be able to apply their knowledge of disaster response to identify the key components of response and recognize appropriate response activities. *To be successful, the participant must be able to correctly recognize the components of disaster response and select the appropriate activities for each response component*.

#### TO2.4 – (Recovery)

Given a critical event scenario, the participant will be able to apply their knowledge of disaster recovery to identify the key components of recovery and recognize appropriate recovery activities. *To be successful, the participant must be able to correctly recognize the components of disaster recovery and select the appropriate activities for each recovery component*.

### Competency 3

#### Demonstrate critical event safety principles

A critical response component for healthcare workers is the ability to protect themselves during a disaster event who in turn will protect the facility and its resources. This encompasses elements of self and scene safety, security issues related to potentially large numbers of victims and contamination, as well as other risks or imminent threats.

#### TO3.1 – (Safety)

Given a critical event scenario, the participant should be able to demonstrate knowledge of critical safety principles by identifying safety threats and appropriate actions. *To be successful the participant must be able to correctly identify appropriate responses to safety threats*.

#### TO3.2 – (Security)

Given a critical event scenario, the participant should be able to demonstrate knowledge of security principles by identifying security threats and appropriate actions. *To be successful the participant must be able to correctly identify appropriate responses to security threats*.

### Competency 4

#### Understand the institutional emergency operations plan

Familiarity with the institutional emergency operations plan is essential for individuals working within the institution to support and implement an effective, coordinated course of action during any critical event.

#### TO4.1 – (Purpose)

The participant should be able to identify the purpose and components of an EOP in critical event response. To be successful, the participant must be able to correctly identify the purposes of the EOP and its components.

#### TO4.2 – (Components)

Given an institutional scenario, the participant will be able to apply knowledge of critical event planning to outline an institutional EOP. *To be successful, the participant must be able to identify the necessary EOP components and functions for the scenario presented*.

### Competency 5

#### Demonstrate effective critical event communications

Communication is a vital element to a successful critical event response. Healthcare workers need to recognize how poor communication can undermine response effectiveness and learn effective critical event communication skills.

#### TO 5.1 – (Communication Overview)

Given a critical event scenario, the participant should be able to apply knowledge of communications to fulfill basic communication needs including identification of appropriate timing, content, recipients, and modalities. *To be successful, the participant must correctly identify the appropriate communication steps, information to be reported, correct reporting authority, and alternative modalities*.

#### TO5.2 – (Communication Implementation)

Given an institutional scenario, the participant should be able apply knowledge of communications to outline a communications plan. *To be successful, the participant must outline the complex communication needs for a critical event*.

### Competency 6

#### Understand the incident command system and your role in it

Effective critical event response requires successful integration of internal and external (local, state, and federal) participants. A recognizable and unified command and control structure is essential to achieve this.

#### TO6.1 – (ICS Overview)

Given a critical event scenario, the participant will be able to recognize their role in the incident command system and identify the corresponding responsibilities and limits of their authority. *To be successful, the participant should be able to identify ICS defined individual tasks and scope of responsibility*.

#### TO6.2 – (ICS Implementation)

Given a simulated incident command scenario, the participant should be able to apply knowledge of the incident command system to outline a command structure and interpret incoming information to make appropriate command decisions. *To be successful, the participant should be able to correctly identify lines of authority, recognize data needs and select appropriate command decisions*.

### Competency 7

#### Demonstrate the knowledge and skills needed to fulfill your role during a critical event

Healthcare workers responding to a critical event require specific knowledge and skills. These encompass triage, personal protection, decontamination, and treatment. Injury pattern recognition and syndrome recognition that suggests the use of particular agents is essential to correct response.

#### TO7.1 – (Triage Skills)

Given an institutional scenario and descriptions of mock victims, the participant will be able to apply knowledge and skills concerning disaster triage systems to rapidly assign victims to appropriate triage categories. *To be successful, the participant must recognize the appropriate triage system and correctly assign triage levels to all victims within a given timeframe*.

#### TO7.2 – (PPE Skills)

Given a critical event scenario and descriptions of mock victims in a simulation, the participant will be able apply knowledge and skills concerning personal protective equipment (PPE) to successfully select, don, and monitor the appropriate level of PPE. *To be successful, the participant must recognize levels of PPE as well as correctly choose and simulate utilization of the appropriate level of protection*.

#### TO7.3 – (Decontamination Skills)

Given a critical event scenario, the participant will be able to apply knowledge and skills regarding decontamination to select, demonstrate, and monitor the correct method(s) of decontamination. *To be successful, the participant must correctly identify the decontamination level as well as select and demonstrate appropriate decontamination techniques*.

#### TO7.4 – (Diagnosis/Treatment)

Given a description of a patient presentation in a critical event scenario, the participant will be able to apply knowledge and skills regarding critical event diagnosis and treatment to identify critical event related syndromes and causative agents and select the appropriate treatment(s). *To be successful, the participant must correctly diagnose the presented clinical syndrome, identify the causative agent and select the appropriate treatment(s)*.

## Discussion

One of the highest priorities identified by the disaster response community in recent years has been to "develop standards and guidelines for education and training in the multi-disciplinary health response to major events that threaten the health status of a community" [31]. The need for rapid and effective disaster training of healthcare staff at all levels is now widely recognized and mandated by the Joint Commission on Accreditation of Healthcare Organizations (JCAHO) [32]. However, differences among healthcare workers such as cultural and educational backgrounds, prior training, work experience and abilities all contribute significant challenges to the standardization of disaster training and education. Training requirements for each job description requires appropriate modification of educational content. Furthermore, effective disaster response demands that selected healthcare workers be equipped not only with knowledge but specific technical skills and decision-making abilities. A competency based approach provides the framework to conduct this type of flexible training.

Competency based approaches to training have been widely implemented and have gained acceptance in medical education in recent years. The advances of competency based education have been well described in the literature [33-38]. For example, the Accreditation Council for Graduate Medical Education (ACGME) has implemented core competency projects to facilitate physician training in specific knowledge and skills. Accordingly, this model has been endorsed by a number of organizations including the American Board of Medical Specialties [33]. This trend reflects an increased focus on curriculum design, establishment of measurable outcomes, adequacy of training, and program evaluation.

The application of competency based education for training healthcare workers in disaster response and other public health emergencies is a relatively new concept. Given the rapidly developing nature of the disaster field, several competency sets have recently become available since this process was conducted. For example, the Columbia School of Nursing, Center for Health Policy and the National Center for Disaster Preparedness at the Columbia University Mailman School of Public Health have described competencies for hospital workers, hospital leaders, clinicians, and nurses as part of a broader competency development program that began with the competencies for public health workers [7]. While competency statements for public health workers, medical students, and various hospital staff in emergency preparedness have been previously outlined, important differences exist in the derivation methods, degree of inclusiveness in development processes, level of detail and how the term competency is defined [25-30]. However, to our knowledge, this is the first set of cross-cutting competencies with specific measurable objectives derived using evidence-based consensus building techniques that addresses the training of all healthcare workers in disaster response. To this end, we have incorporated a systematic review of all existing related competencies and peer-reviewed literature, nationally recognized disaster courses, educational theory and expert panel consensus.

Deriving training content from a single set of cross-cutting competencies offers both theoretical and practical advantages over utilization of distinct competency sets for each type of healthcare worker being trained. First, the multidisciplinary nature of critical event preparedness and response and the importance of an integrative approach to training can not be overstated. All healthcare workers require training to develop the fundamental knowledge and skills to function independently but also to act in part of a coordinated response effort. Cross-cutting competencies unite the common underpinnings of requisite training for all healthcare workers; a departure from historically discipline-focused training.

Given the importance of problem solving and decision-making skills, the cross-cutting competencies and terminal objectives are designed to emphasize higher levels of learning such as synthesis and performance, rather than strictly knowledge acquisition [13]. In accordance with educational theory, each terminal objective was constructed so that performance may be readily tested or evaluated [13-15]. Each terminal objective can be easily mapped to the most appropriate target audience(s), with different target audiences receiving the content that will allow that group to best fulfill the relevant competency statement as applied to their job. For example, critical event leadership has different training needs from front-line physicians who in turn have different training needs from community-based practitioners. Thus, while cross-cutting competencies apply to all healthcare workers, certain terminal objectives such as those related to PPE and decontamination skills deemed essential for first receivers would be unlikely required of healthcare administrators.

Future steps will include application of this structured evidence-based process to further divide each terminal objective into enabling objectives, a series of clear statements of component knowledge, skills and/or attitudes, each with easily measurable and well-defined performance characteristics.

## Conclusion

The cross-cutting competencies and accompanying objectives developed using this systematic evidence-based consensus building approach may serve as a paradigm for healthcare worker disaster training and education. While reaching similar general conclusions to other competency work that has been conducted, this framework offers the opportunity for greater standardization in training and evaluation by facilitating a uniform approach to derivation of objectives, content and evaluation.

## Competing interests

There are no situations with this manuscript that may be perceived as a conflict of interest or as a copyright constraint. If accepted for publication, permissions to reproduce published material or personal communications in all forms and media are granted to BioMed Central.

## Authors' contributions

Each author has participated in the preparation of this manuscript. (EBH contributed in study concept and design, analysis and interpretation of the data, drafting of the manuscript, critical revision of the manuscript and obtaining funding, TLT contributed in study concept and design, analysis and interpretation of the data, drafting of the manuscript and critical revision of the manuscript, EBB contributed in study concept and design, analysis and interpretation of the data, and critical revision of the manuscript, DW contributed in acquisition of the data, analysis and interpretation of the data, and administrative and material support, GDK contributed in study concept and design, obtaining funding, administrative and technical support and study supervision, GBG contributed in study concept and design, analysis and interpretation of the data, drafting of the manuscript, critical revision of the manuscript, obtaining funding and study supervision.)

## Pre-publication history

The pre-publication history for this paper can be accessed here:



## Supplementary Material

Additional File 1 Additional references from the literature search and structured review of existing courses and competencies which are not cited in the text are included. Click here for file

## Figures and Tables

**Figure 2:**
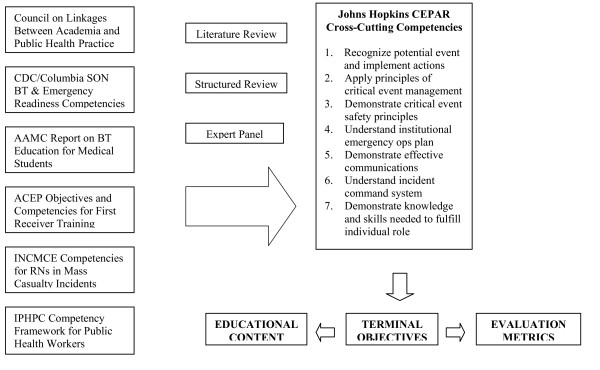
Development of cross-cutting competencies.
