# Supplementary material for: Healthcare worker competencies for disaster training
Source: BMC Med Educ. 2006 Mar 20;6:19. doi: 10.1186/1472-6920-6-19 (PMC1471784; doi:10.1186/1472-6920-6-19)
Supplement: Additional File 1 — Additional references from the literature search and structured review of existing courses and competencies which are not cited in the text are included. [file 1472-6920-6-19-S1.doc]

**Additional references**

Adams K. The South Carolina nurses association unveils preliminary findings of the nursing school survey on competencies for entry-level registered nurses related to mass casualty incidents. S.C. Nurse. 2005; 12(4):1.

Alexander AJ, Bandiera GW, Mazurik L. A multiphase disaster training exercise for emergency medicine residents: Opportunity knocks. Acad Emerg Med. 2005; 12(5):404-409.

Agency for Toxic Substances and Disease Registry. A primer on health risk communication principles and practices. (http://HREF="http://www.atsdr.cdc.gov/HEC/primer.html") Accessed on May 20, 2004.

American Academy of Family Physicians. Educational resources on WMD preparedness and response. (http://HREF="http://www.aafp.org/btresponse.xml") Accessed on May 20, 2004.

American Academy of Pediatrics Children, Terrorism , and Disasters.

(http://HREF="http://www.aap.org/terrorism/index.html") Accessed on May 20, 2004.

American Association for Health Education, National Commission for Health Education Credentialing, Society for Public Health Education. (1999). A competency-based framework for graduate-level health educators. Washington, D.C.

American Association of Colleges of Nursing. (1998) The essentials of baccalaureate education for professional nursing practice. Washington, DC

American College of Physicians. HREF="http://www.acponlineorg/bioterro/index.html" WMD preparedness and response

American College of Surgeons. HREF="http://www.facs.org/civiliandisasters/intro.html" Disasters from Biological and Chemical Terrorism – What Should the Individual Surgeon Do? A Report from the Committee on Trauma and Statement on Unconventional Acts of Civilian Terrorism: A Report from the Board of Governors.

American Medical Association HREF="http://www.ama-assn.org/go/DisasterPreparedness" JAMA articles relating to WMD preparedness and response

American Nurses Association. (2002) Position Statement on Work Release during a Disaster: Guidelines for Employers. Adopted by the ANA Board of Directors. Effective June 24, 2002

American Psychiatric Association HREF="http://www.psych.org/pract_of_psych/disaster_psych.cfm" Preparing for and Responding to Disasters and Other Traumatic Events.

American Public Health Association HREF="http://www.apha.org/united/" WMD preparedness and response from a public health perspective.

American Society of Microbiology HREF="http://www.asmusa.org/pasrc/bioprep.htm" Articles and reports associated with biologic agents.

Andress K. A postevent smallpox mass vaccination clinic exercise. Disaster Manag Response. 2003; 1(2):54-58.

Association of Medical School Microbiology and Immunology Chairs HREF="http://www.amsmic.org/educational_links.html" A Study of the opinions of microbiology chairs with regard to teaching bioterrorism related topics to medical students.

Association of State and Territorial Health Officials HREF="http://www.astho.org/index.php?template=1bioterrorism.html" WMD Preparedness and Response from the Perspective of State Health Departments

Association for Professionals in Infection Control and Epidemiology HREF="http://www.apic.org/bioterror/" WMD Educational Resources from Government Agencies and Professional Societies

Barbera, J. & Macyntire, A. (2003) Jane’s mass casualty handbook: Hospital emergency preparedness and response. Surrey, UK: Jane’s Information Group, Ltd.

Barnett DJ, Everly GS, Parker CL, Links JM. Applying educational gaming to public health workforce emergency preparedness. Am J Prev Med. 2005;28(4):390-395.

Beaton RD, Johnson LC. Instrument development and evaluation of domestic preparedness training for first responders. Prehospital Disaster Med. 2002; 17(3):119-125.

Beaton RD, Stevermer A, Wicklund J, Owens, Boase J, Oberle MW. Evaluation of the Washington State National Pharmaceutical Stockpile dispensing exercise, part II- dispensary site worker findings. J Public Health Manag Pract. 2004; 10(1):77-85.

Bernardo LM, Kaspar P. Pediatric implications in bioterrorism: Education for healthcare providers. Disaster Manag Response. 2003; 1(2):52-53.

Biological and Chemical Terrorism: Strategic Plan for Preparedness and Response, MMWR April 21, 2000/49(RR04); 1-14

Bond EF, Beaton R. Disaster nursing curriculum development based on vulnerability assessment in the pacific northwest. Nurs Clin North Am. 2005; 40(3):441-51, viii.

BT and Emergency Readiness Competencies. http://cpmcnet.columbia.edu/dept/nursing/institutes-centers/chphsr/btcomps.html

CDC BT Event Response Operational Plan. (in development, August 2000). Bioterrorism Response and Preparedness Program, NCID, CDC. Scott Lillibridge, M.D., MPH, Director.

Center for Civilian Biodefense Strategies. HREF="http://www.hopkins-biodefense.org/index.html"

Ciottone GR, Old A, Nicholas S, Anderson PD. Implementation of an emergency and disaster medical response training network in the commonwealth of independent states. J Emerg Med. 2005; 29(2):221-229.

COL Core Competencies. The Council on Linkages between Academia and Public Health Practice. http://www.trainingfinder.org/competencies/

Cole, F. & Ramirez, E. (1999). Evaluating an emergency nurse practitioner education program for its relevance to the role. Journal of Emergency Nursing, 25(6), 547-550

Cole FL. The role of the nurse practitioner in disaster planning and response. Nurs Clin North Am. 2005; 40(3):511021, ix.

Competencies for Public Health Workers: a collection of competency sets. http://www.phppo.cdc.gov/owpp/docs/compgrid0829.pdf

Core Legal Competencies for Public Health Professionals. http://www.publichealthlaw.net/Training/TrainingPDFs/PHLCompetencies

Covich JR, Parker CL, White VA. The practice community meets the ivory tower: A health department/ academic partnership to improve public health preparedness. Publi Health Rep. 2005;120 Suppl 1:84-90.

Darling RG, Eitzen EM, Mothershead JL and Waeckerie JF, ed. May 2002 Emergency Medicine Clinics of North America: Bioterrorism. WB Saunders and Co: Philadelphia, PA, 2002.

Davies K, Deeny P, Raikkonen M. A transcultural ethos underpinning curriculum development: A master’s programme in disaster relief nursing. J Transcult Nurs. 2003; 14(4):349-357.

Davies K, Moran L. Nurses need advanced skills in disaster health care. Br J Nurs. 2005; 14(4):190.

DiMaggio C, Markenson D, Redlener I. Prepareing for disasters: What you should know, and when should you know it? JAAPA. 2005; 18(3):40-3, 45, 48 passim.

Durch, J.S. et al. (Eds.) (1997). Improving health in the community: a role for performance monitoring. Washington, D.C.: National Academy Press

Edwards AG, Donaldson O, Walsh E, Karantana A. Medical staff need to be aware of major incident planning. BMJ. 2003; 326(7392):762.

Emergency Preparedness and Response Competencies for Hospital Workers. <http://cait.cpmc.columbia.edu:88/dept/sph/CPHP/hospcomps/pdf>

Emergency Response Clinician Competencies in Initial Assessment and Management. http://www.atpm.org/education/ClinicianCompetencies.pdf.

Environmental Health Competencies. http://www.apha.org/ppp/ehproject.htm.

Estrada LC, Fraser MR, Cioffi JP, et al. Partnering for preparedness: The project public health ready experience. Public Health Rep. 2005; 120 Suppl 1:69-75.

Federal Emergency Management Agency HREF="http://www.fema.gov/" Emergency Management Systems

Food and Drug Administration HREF="http://www.fda.gov/oc/opacom/hottopics/bioterrorism.html" FDA Counter-terrorism initiatives and information on food safety

Fothergill A, Palumbo MV, Rambur B, Reiner K, McIntosh B. The volunteer potential of inactive nurses for disaster preparedness. Public Health Nurs. 2005; 22(5):414-421.

Fraser, M. & Fisher, V.S. (January 2001). Elements of effective bioterrorism preparedness: A planning primer for local public health agencies. Washington, DC: National Association of County and City Health Officials.

Gable, C.B. (1990). A compendium of public health data sources. American Journal of Epidemiology, 131, 381-94

Galligan JM. Dentists can contribute expertise in a major public health disaster. J Calif Dent Assoc. 2004; 32(8):701-708.

Hagen JF, Jr, American Academy of Pediatrics Committee on Psychosocial Aspects of Child and Family Health, Task Force on Terrorism. Psychosocial implications of disaster or terrorism on children: A guide for the pediatrician. Pediatrics. 2005; 116(3):787-795.

Halverson, P.K. (2000). Performance Measurement and Performance Standards: old wine in new bottles. Journal of Public Health Management and Practice 6:5, vi-x.

Handbook on training and the examination for membership regulations. London: Faculty of Public Health Medicine, 1997.

Hennessy, C.H. et al. (1994). Measuring health-related quality of life for public health surveillance. Public Health Reports, 109(5), 665-672.

Hick JL, Penn P, Hanfling D, Lappe MA< O’Laughlin D, Burtein JL. Establishing and training health care facility decontamination teams. Ann Emerg Med. 2003; 42(3):381-390.

Hoard ML, Tosatto RJ. Medical reserve corps: Strengthening public health and improving preparedness. Disaster Manag Response. 2005; 3(2):48-52.

Horney JA, Sollecito W, Alexander LK. Competency-based preparedness training for public health practitioners. J Public Health Manag PRact. 2005; 11(6 Suppl):S147-S149.

Infectious Diseases Society of America HREF="http://www.idsociety.org/BT/ToC.htm" Educational materials associated with bioterrorism agents.

Informatics Competencies for Pubilc Health Professionals. http://www.nwcphp.org/phi/comps.

Journal of Public Health Management and Practice. Aspen Publication

Kingdon, J.W. (1995). Agendas, alternatives, and public policies (2nd ed.) Boston: Little, Brown.

Kisely, S.R., Donnan, S.P. Competencies for Part II of the examination for membership of the Faculty of Public Health Medicine. J. Public Health Medicine, 1997; 19(1): 11-17

Knobler SL, Mahmoud AAF, and Pray, LA, ed. Biological Threats and Terrorism: Assessing the Science and Response Capabilities. National Academy Press: Washington, DC, 2002.

Krieger, N. et al. (1997). Can we monitor socioeconomic inequalities in health? A survey of health departments’ data collection and reporting practices. Public Health Reports; 112, 481-91.

Madge SN, Kersey JP, Murray G, Murray JR. Are we training junior doctors to respond to major incidents? A survey of doctors in the wessex region. Emerg Med J. 2004; 21(5):577-579.

Moore, C.M. (1987) Group techniques for idea building. Newbury Park, CA: Sage Publications.

Moore, F.I. (September 1999). Functional Job analysis: Guidelines for task analysis and job design. Geneva: World Health Organization

National Association of County and City Health Officials HREF="http://www.naccho.org/NACCHO-RespondsToBT.cfm" WMD preparedness and response from the perspective of local health departments

National Library of Medicine. HREF="http://www.nlm.nih.gov/medlineplus.biodefenseandbioterrorism.html"

Ng AT. Cultural diversity in the integration of disaster mental health and public health: A case study in response to bioterrorism. Int J Emerg Ment Health. 2005; 7(1):23-31.

Niska RW, Burt CW. Bioterrorism and mass casualty preparedness in hospitals: United States, 2003. Adv. Data. 2005; (364):1-14.

Novick LF, Marr JS, ed. Public Health Issues in Disaster Preparedness: Focus on Bioterrorism. Aspen Publishers, Inc.: New York, NY, 2001

Olness K, Sinha M, Herran M, Cheren M, Pairojkul S. Training of health care professionals on the special needs of children in the management of disasters: Experience in Asia, Africa, and Latin America. Ambul Pediatr. 2005; 5(3):244-248.

Orton S, Umble K, Davis MV, Porter JE. Disasters and bioterrorism: Does management training develop readiness? Pub Health Rep. 2002; 117(6):596-598.

Parker CL, Barnett DJ, Fews AL, Blodgett D, Links JM. The road map to preparedness: A coimpetency-based approach to all-hazards emergency readiness training for the public health workforce. Public Health Rep. 2005; 120(5):504-514.

Petersen, D.J. & Alexander, G.R. (2001). Needs assessment in public health: a practical guide for students and professionals. NY: Kluwer Academic/Plenum Publishers

Post TOPOFF Action Plan. Phase I – Improving CDC’s Immediate Response Capabilities.

The Public Health Competency Handbook. http://www.populationhealthfutures.com/handbook.htm

Public Health Nursing Competencies. http://www.uncc.edu/achne/quadcouncil/Final_PHN_Competencies.doc

Rega P. Disaster medical education for all physicians and physician extenders. Ann Emerg Med. 2000; 35(3):314-316.

Rogers, E.M. (1995) Diffusion of Innovations (3rd ed.). New York: Free Press.

Roush, S., et al. (1999). Mandatory reporting of diseases and conditions by health care professionals and laboratories. Journal of the American Medical Association, 202(2), 164-70.

Sarpy SA, Chauvin SW, Anderson AC. Evaluation of effectiveness of the south central center for public health preparedness training. Public Health Rep. 2003; 118(6):568-572.

Sarpy SA, Warren CR, Kaplan S, Bradley, Howe R. Simulating public health response to a severe acute respiratory syndrome (SARS) event event: A comprehensive and systematic approach to designing, implementing and evaluating a tabletop exercise. J Public Health Manag Pract. 2005; 11(6 Suppl):S75-S82.

Schliepman AR, Gerbaudo VH, Castronova FP, Jr. Radiation disaster response: Preparation and simulation experience at an academic medical center. J Nucl Med Technol. 2004; 32(1):22-27.

Schreiber S, Yoeli N, Paz G, et al. Hospital preparedness for possible nonconventional casualties: An Israeli experience. Gen Hosp Psychiatry. 2004; 26(5):359-366.

Society for Healthcare Epidemiology of America HREF="http://www.shea-online.org/BTprep.html" Bioterrorism-related clinical issues

Stanley JM. Disaster competency development and integration in nursing education. Nurs Clin NorthAM. 2005; 40(3):453-67, viii.

Stephens, I., Arora, S. Hurdling for beginners – A specialist registrar guide to passing MFPHM. http://fester.his.path.cam.ac.uk/phealth/exams.htm

Summary Report: Bioterrorism Preparedness and Response Program, Education and Training Constituents Meeting, December 23, 1999, Centers for Disease Control and Prevention, Atlanta, GA.

Task Force of Health Care and Emergency Services Professionals on Preparedness for Nuclear, Biological, and Chemical incidents. (April 2001) Final Report: Developing objectives, content, and competencies for the training of emergency medical technicians, emergency physicians, and emergency nurses to care for casualties resulting from nuclear, biological, or chemical incidents (Contract No. 282-98-0037). American College of Emergency Physicians.

Task Force on Public Health Workforce Development. CDC/ATSDR Strategic Plan for Workforce Development. Atlanta. GA: Centers for Disease Control and Prevention, Agency for Toxic Substances and Disease Registry; 1999

Taylor, H. (1997). Public Health: Two words few people understand even though almost everyone thinks public health functions are very important. New York: Louis Harris and Associates

Thorne CD, Oliver M, Al-Ibrahim M, Gucer PW, McDiarmid MA. Terrorism preparedness training non-clinical hospital workers: Tailoring content and presentation to meet workers’ needs. J Occup Environ Med. 2004; 46(7):668-676.

The MFPHM Part II Abstracts Database. London: Faculty of Public Health Medicine, 1999. http://195.224.168.94/

Turnock, BJ (1997). Public Health: What it is and how it works. Gaithersburg, MD: Aspen Publishers, Inc.

Turnock BJ. Roadmap for public health workforce preparedness. J Public Health Manag PRact. 2003;9(6):471-480.

UPI News Article: U.S. totally unprepared for bioterrorism, Tuesday, 22 August 2000.

Uniformed Services University of the Health Sciences HREF="http://www.usuhs.mil/" Medical Management of Radiological Casualties Handbook, Second Edition. April 2003

Uniformed Services University of the Health Sciences Graduate School of Nursing. (November 2001) Materials and personal communication from Faye G. Abdellah, Dean and Professor, Graduate School of Nursing, including examples of advanced practice nursing objectives, course descriptions, and course offerings.

United States Air Force. (2001). RSV for AFSC46XX and RSV for AFSC 46N3E. Competencies for all clinical and emergency department nurses. (Unpublished documents) Washington, DC

University of Ulster, School of Health Sciences, Nursing. (1998) Course documents for postgraduate diploma/MSc in disaster relief nursing for entry September 1999. Ulster, UK

U.S. Army Medical Research Institute of Infectious Diseases HREF="http://www.usamriid.army.mil/education/bluebook.html" Medical Management of Biologic Casualties Handbook.

U.S. Army Medical Research Institute of Chemical Defense HREF="http://ccc.apgea.army.mil/products/handbooks/books.htm" Medical Management of Chemical Casualties Handbook.

U.S. Department of Health and Human Services, Centers for Disease Control and Prevention, Public Health Practice Program Office, National Public Health Performance Standards Program.

Veenema TG. Chemical and biological terrorism preparedness for staff development specialists. J Nurses Staff Dev. 2003; 19(5):218-25.

Verklan MT> Be prepared: How to establish competency before disaster strikes. Patient Care Manag. 2002; 18(1):1, 10-1.

Virtual Naval Hospital HREF="http://www.vnh.org/Providers.html#NBC" Information on Biological, Chemical, and Nuclear Warfare and Radiation Safety.

Walker BL, Harrington SS. Can nursing facility staff with minimal education be successfully training with computer-based training? Nurs Educ Today. 2004; 24(4):301-309.

Water Health Connection HREF="http://www.waterhealthconnection.org/" Recognizing Waterborne Disease and the H ealth Effects of Water Pollution (Including a section entitled: Physician Preparedness for Acts of Water Terrorism)

Weiner E, Irwin M, Trangenstein P, Gordon J. Emergency preparedness curriculum in nursing schools in the United States. Nurs Educ Perspect. 2005; 26(6):334-339.

Welling L, Perez RS, can Harten SM, et al. Analysis of the pre-incident education and subsequent performance of emergency medical responders to the Volendam café fire. Eur J Emerg Med. 2005; 12(6):265-269.

Wolf, R.M. (1990) Evaluation in Education: Foundations of competency assessment and program review. Pg. 54 (3rd ed.) New York, NY: Praeger Publishers

World Health Organization. (1999) Development of a disaster preparedness tool kit for nursing and midwifery: Report on a WHO meeting held at the University of Ulster 20-21st August 1999. Copenhagen, Denmark: WHO.
